# Influence of Post-Core and Crown Type on the Fracture Resistance of Incisors Submitted to Quasistatic Loading

**DOI:** 10.3390/polym13071130

**Published:** 2021-04-02

**Authors:** Sadullah Uctasli, Yakup Boz, Sercan Sungur, Pekka K. Vallittu, Sufyan Garoushi, Lippo Lassila

**Affiliations:** 1 Department of Prosthodontics, Faculty of Dentistry, University of Ankara, 06790 Ankara, Turkey; sadullah.uctasli@dentistry.ankara.edu.tr (S.U.); dt.yakup.boz@outlook.com (Y.B.); sercan.sungur@dentistry.ankara.edu.tr (S.S.); 2 Turku Clinical Biomaterial Center-TCBC, Department of Biomaterials Science, Institute of Dentistry, University of Turku, 20500 Turku, Finland; pekval@utu.fi (P.K.V.); sufgar@utu.fi (S.G.); 3 City of Turku Welfare Division, Oral Health Care, 20101 Turku, Finland

**Keywords:** fracture behavior, post-core, CAD/CAM, fiber composite

## Abstract

The aim of this paper was to evaluate the fracture resistance and failure type of maxillary incisor teeth, rebuilt with various types of post-core restorations and full crowns made of either direct conventional particulate filler composite (PFC, G-aenial Anterior, GC, Tokyo, Japan) or indirect CAD/CAM restorations (composite Cerasmart 270 and glass ceramic LiSi Block from GC). One hundred (*n* = 10/group) central incisors were cut and divided into 10 experimental groups restored with different approaches. In approach A, teeth were restored with a core build-up composite (Gradia Core, GC) for a core and full crown of PFC. Approach B had teeth restored using composite core and prefabricated fiber posts, and a complete crown of either PFC or CAD/CAM. Approach C contained teeth restored with a core of short fiber-reinforced composite (everX Flow, GC) and prefabricated fiber posts, and a complete crown of either PFC or CAD/CAM. In approach D, the teeth had a core of short fiber-reinforced composite only, and a complete crown of either PFC or CAD/CAM restorations. The root canals were prepared, and when posts were used, they were luted with either a dual-cure resin cement (LinkForce, GC) or everX Flow. As the control, sound teeth (*n* = 10) were used. Restorations were quasi-statically loaded until fracture. Failure type was visually investigated. The interface between the fiber post and luting cement was investigated using SEM, before and after completion of the loading test. The data were analyzed by analysis of variance (*p* = 0.05) followed by Tukey’s test. None of the restorative approaches restored the fracture load strength of intact teeth (*p* < 0.05). Restorations with additional fiber posts (Approaches B and C) had higher load-bearing capacity (*p* < 0.05) than restorations without fiber posts (Approaches A and D). Restorations that had short fiber-reinforced composite cores with or without fiber posts presented more repairable failures. Using short fiber-reinforced composite as post-luting and core build-up material with conventional fiber posts proved to be a promising method to strengthen severely damaged incisors.

## 1. Introduction

In order to have good retention for full crown restoration, damaged root canal treated (RCT) teeth usually need significant build-up with different post-core materials [1]. The sum of the residual coronal structure and internal root structure defines the needs of post-usage. The main objective of an endodontic post is to give retention when the remaining tooth structure is not enough to retain the core restoration [2]. When less than half of the crown remains in the anterior teeth, post placement is recommended [3,4]. The presence or absence of 1.5–2 mm-high coronal dentin after preparation, known as the “ferrule,” is one of the main factors affecting the performance of post-restorations [5]. The ferrule’s aim is to redistribute the stress that occurs on the root’s outer coronal third, potentially changing the fracture pattern to one that can be repaired [5]. Previous studies have concluded that RCT teeth after post-core build-up should be restored with indirect crowns [6,7], although others have shown little difference between various types of crown restorations [8,9]. Owing to the contrary findings from many in vitro studies, there are still no formal instructions or agreements on the selection of final crown restorations [10]. Various forms of fiber posts have been presented recently to afford the dental discipline a substitute to prefabricated or casted metal posts for restoration of RCT teeth; since the modulus of elasticity of these fiber posts is similar to that of dentin, they are a better alternative than metal posts [11].

It should be noted that anterior teeth rebuilt with a post have a three times higher fracture rate than posterior teeth [12]. This may be justified by the higher horizontal forces that these teeth are subjected to as a result of their location in the arch [13]. The post–root canal interface is challenged when greater horizontal forces are present, and any possible fault will later lead to failure. [13]. With the fiber posts, the most frequent types of failure are loss of retention or post fracture [14]. The explanations are various, including the root weakening during post space adjustment, the post’s inaccurate fit due to the irregular geometry and cross section of the root canal, or the post’s failure to properly bind to the luting or core build-up material [13]. The amount and adaptation of the fiber post in the critical cervical part of the tooth, according to Vallittu, could determine the effectiveness of restorative procedures involving post insertion [15]. When the post fails to accommodate tightly, especially at the coronal third of the root, the resin cement film becomes thick, allowing voids to form and cracks during loading, which can lead to post debonding [16]. Fabricating an individualized fiber post from multiple unidirectional fiber-reinforced composites is one way to solve this issue [17,18]. Another alternative is to make the post and core build-up directly within the root canal from short fiber-reinforced composites (SFRCs) [19,20,21,22]. In this “bioblock” construction method, packable and flowable SFRCs are used to fill both the root canal space and the coronal cavity in 4–5 mm thick horizontal increments [21,23,24].

However, challenges are presented as to whether it is possible or not to use the flowable SFRC for post-core build-up, or for luting conventional fiber posts to rebuild RCT anterior teeth in the presence of ferrule. To the best of the authors’ understanding, this has not been extensively studied in the literature. Therefore, the aim of this laboratory research was to assess the load-bearing capacity and failure types of RCT anterior teeth restored with various fiber-reinforced post-core composites and full crowns. The null hypotheses were that (1) the teeth restored with the investigated restorative approaches would have similar load-bearing capacity, and that (2) the type of failure will be unaffected by the restorative technique used.

## 2. Materials and Methods

Table 1 lists all of the materials used in this study.

**Figure 1:**
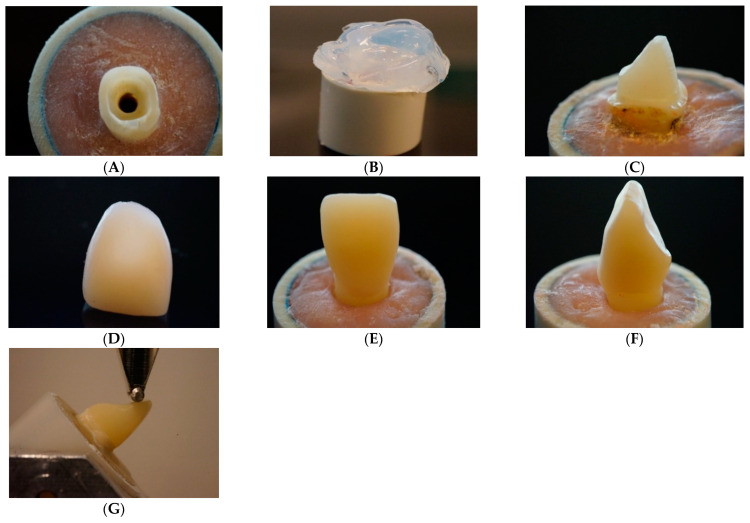
(**A**) tooth preparation, (**B**) transparent template mold for composite build-up, (**C**) post and core restoration, (**D**) crown fabrication, (**E**,**F**) labial and proximal views of final crown restoration, (**G**) inclined quasistatic-load test setup.

In total, 110 intact human maxillary central incisors with 14 mm root lengths and equivalent mesiodistal and buccolingual measurements were selected. The extracted teeth were collected (University of Ankara, Ankara, Turkey) and subsequently placed in sodium hypochlorite (5.25%) for a few minutes before being preserved in a saline solution at 4 °C for up to 6 months before being used. The research and ethics committee of the University of Ankara, Faculty of Dentistry, accepted the procedure for using these teeth for this research (12 July/7 January 2020). Only teeth with a maximum deviation of 10% from the determined mean were included in this study based on the measurements. All crowns (*n* = 100) were horizontally sectioned using a diamond disk with water cooling 2 mm above the cementoenamel junction (CEJ). All teeth were prepared by two trained operators. The pulp was removed and the periodontal tissue was cleaned. Under water cooling, post space preparations were made with post drills (Parapost stainless drills, Coltène/Whaledent, Mahwah, NJ, USA). The teeth were then mounted just 1 mm below the CEJ on an acrylic block (Ø 2.5 cm) using self-cure acrylic resin (Palapress; Heraus Kulzer, Wehrheim, Germany) (Figure 1A).

**Figure 2:**
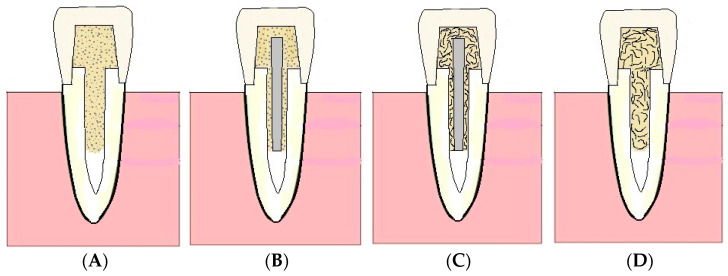
Diagram showing the restorative approaches with various post-core restorations. Approach (**A**) Gradia Core as post-core and complete crown of PFC. Approach (**B**) fiber post and Gradia Core to build up core and complete crown of either PFC or CAD/CAM. Approach (**C**) fiber post and core made of SFRC and complete crown of either PFC or CAD/CAM. Approach (**D**) SFRC as post-core and complete crown of either PFC or CAD/CAM.

Next, post-core and crowns were made based on four different restorative approaches (Figure 2) subdivided into 10 groups (*n* = 10/group), presented in Table 2. A total of ten sound teeth were left as control specimens (Group 11).

### 2.1. Post and Core Fabrication

The teeth’s coronal surfaces were etched for 20 s with a 37% phosphoric acid etch-gel (Scotchbond, 3M ESPE, St. Paul, MN, USA), then rinsed, and air-dried gently. Dentin adhesive was applied in compliance with the manufacturer’s guidance (Scotchbond Universal, 3M ESPE, Irvine, CA, USA). A dual cure activator (DCA) and adhesive were mixed together (1:1) and applied as intracanal dentin adhesive. A transparent template matrix (Memosil2, Heraeus Kulzer GmbH, Hanau, Germany) of a well-constructed core was used to assist core fabrication in order to achieve the same core dimensions (Figure 1B).

Composite cores were made and polymerized (20 s per layer) (Elipar TM S10, 3M ESPE, Seefeld, Germany) incrementally, 5 mm incisal to the sectioned tooth surfaces (Figure 1C). The light had a wavelength of 430 to 480 nm and a power irradiance of 1600 mW/cm^2^. For approach A, posts and cores were made of Gradia Core, which was applied and polymerized in bulk into the prepared canals. Cores were fabricated and polymerized as previously mentioned. For approach B, fiber posts were luted with dual-cure luting resin (Gradia Core), which was delivered into the root canal using automix tips. For approach C, fiber posts were luted with SFRC. After surface treatment with a primer (G-Multi Primer, GC Corp, Tokyo, Japan), a prefabricated (1.6 mm) glass fiber post (MI Core Fiber Post) was carefully placed into the luting-filled root canal space. Excess luting material was removed at the level of crown sectioning after the post reached the intended length (12 mm). The luting resin was polymerized for 40 s (Elipar TM S10) at a 45° angle. The fiber posts were 4 mm extended above the coronal surface of the prepared teeth, and cores were fabricated and polymerized as mentioned previously.

For approach D, posts and cores were made of SFRC, which was applied and polymerized in layers (4–5 mm) into the prepared canals, and cores were made and polymerized as previously mentioned.

### 2.2. Crown Fabrication

Final crown fabrication was planned to imitate either the direct fabricated technique with conventional light-cured PFC (G-aenial Anterior) or the indirect technique using Cerasmart 270 and LiSi Blocks with a CAD/CAM device (CEREC, Sirona Dental Systems Inc., Long Island City, NY, USA).

#### 2.2.1. Direct PFC Crowns (Groups 1–4)

A transparent template matrix of an ideally contoured crown was used to aid crown fabrication in order to minimize variations in specimens. The crown mold was fabricated, then filled with PFC, pressed, placed over the build-up core, and light cured from the outside. The light-curing tip was positioned in close proximity to the crown surface (1–2 mm). The crown mold was removed after polymerization.

#### 2.2.2. Indirect CAD/CAM Crowns (Groups 5–10)

Crowns were designed and milled from Cerasmart 270 (Groups 5–7) and LiSi Blocks (Groups 8–10), after a photo impression of the post-core model was taken (Figure 1D). All restorations’ bonded surfaces were acid etched for 60 s with 9.6% hydro-fluoric acid (Pulpdent Corporation, Watertown, MA, USA), then washed and air-dried before cementation. The multi primer (G-Multi Primer) and dual-cure resin cement (G-CEM linkForce) were then used to cement the CAD/CAM-fabricated restorations. After that, a hand light-curing unit (Elipar TM S10) was used from all directions (20 s per segment) and in close contact with the crown surface.

All direct and indirect restorations were polished with abrasive polishing points (Jiffy Polishers, Ultradent, South Jordan, UT, USA) prior to the inclined loading test to ensure that the margin of root and crown material was visible (Figure 1E,F). All of the fabricated specimens were kept in distilled water for 48 h at 37 °C before testing.

### 2.3. Fracture Load Test

A universal testing machine (Lloyd model LRX, Lloyd Instruments Ltd., Fareham, UK) was used to apply a quasi-static load to the restored teeth at a speed of 1 mm/min [1]. The restored tooth with the acrylic block was firmly attached to the inclined metal base to create a 45-degree angle between the loading tip (spherical Ø 2 mm) and palatal surface of the incisal edge (Figure 1G). Each restored tooth’s loading event was recorded until it fractured, and the fracture type for each specimen was visually analyzed and classified into two types of failures. A two-examiner agreement was used to distinguish between repairable and irreparable fractures. A repairable fracture is the type that ends above the CEJ, whereas an irreparable fracture is the type that extends below the CEJ.

### 2.4. Microscopic Analysis of Fiber Post–Cement Interface

Four additional restorations representative of Approaches B and C were fabricated following the aforementioned restorative technique. Specimens (*n* = 4) were sectioned using a ceramic cutting disc spinning at 100 rpm (Secotom-50, Struers, Copenhagen, Denmark) under water cooling before and after the loading test. An automatic grinding machine was then used to gently polish the sectioned tooth using #4000-grit silicon carbide papers at 300 rpm under water cooling (Rotopol-1, Struers, Copenhagen, Denmark). Scanning electron microscopy was used to examine representative specimens (SEM, JSM 5500, Jeol Ltd., Tokyo, Japan). SEM analysis (×500 and 1000 magnification) was performed at an operating voltage of 15 kV, spot-size of 37 and working distance of 18 mm. Prior to observation, all the specimens were gold-coated using a sputter coater in a vacuum evaporator (BAL-TEC SCD 050 Sputter Coater, Balzers, Liechtenstein).

### 2.5. Statistical Analysis

The data were analyzed using analysis of variance (ANOVA) and a Tukey HSD post hoc test in SPSS version 23 (IBM Corp., Armonk, New York, NY, USA). Three-way ANOVA (*p* = 0.05) was used to investigate the differences among the groups. The dependent factor was the load-bearing capacity, whereas the independent factors were the post-core materials, with or without prefabricated fiber post and final crown material. In addition, statistical differences in failure modes were investigated by chi-square tests at a significance level of *p* = 0.05.

## 3. Results

**Figure 3:**
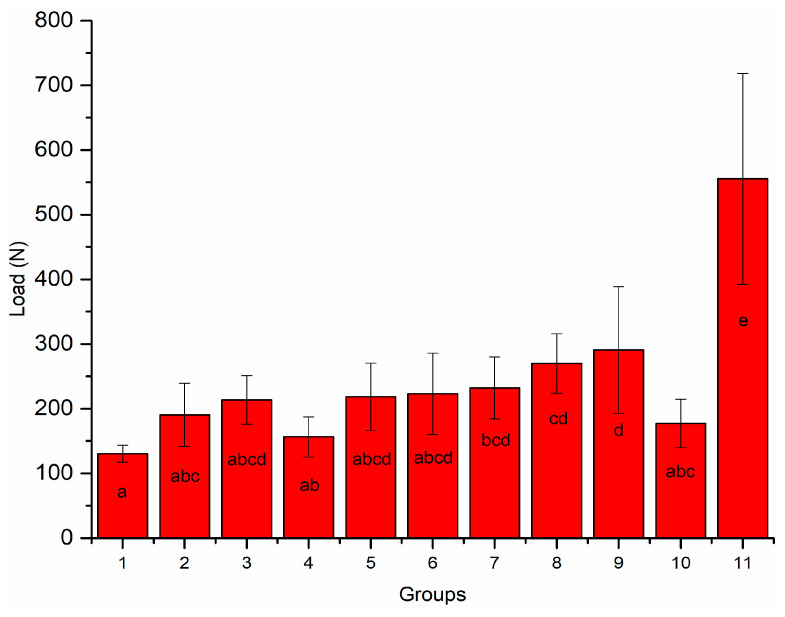
Mean values of load-bearing capacity (N) with standard deviation of tested restorations (Groups 1–10) and intact teeth (Group 11). Groups denoted with the same letters are not statistically different (*p* < 0.05).

Figure 3 shows the load-bearing capacity of teeth restored using various techniques. The restoration technique had a significant impact on the load-bearing capacity (*p* < 0.05), but there was some interaction between the groups. None of the restorative approaches restored the fracture strength of intact teeth (*p* < 0.05). As shown in Figure 3, crown specimens in Group 9, where SFRC (everX Flow) was used for post-luting and core build-up resin, had the highest load-bearing capacities (291 ± 98 N), which were not significantly different (*p* > 0.05) from other Groups (3, 5, 6, 7, and 8). Crowns reinforced with conventional (unidirectional) fiber posts (Approaches B and C) had a higher load-bearing capacity (*p* < 0.05) than crowns without fiber posts (Approaches A and D).

In addition, the data revealed that restorations made of SFRC core had a higher load-bearing capacity than those restored with Gradia as the core material (Figure 3). However, the difference was not statistically significant (*p* > 0.05).

**Figure 4:**
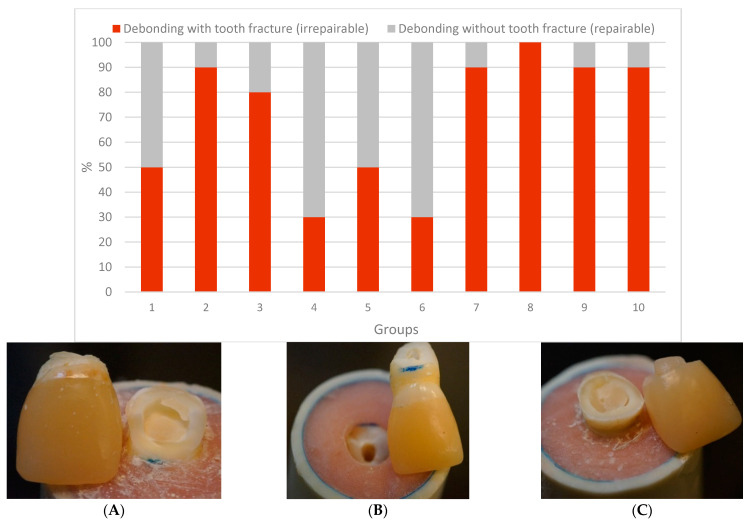
Percentages and photographs of failure modes of the tested restorations. (**A**,**B**) irrepairable fracture type, (**C**) repairable fracture type.

Indirect CAD/CAM-fabricated crowns showed higher load-bearing capacities than direct PFC crowns (*p* < 0.05). No statistically significant differences (*p* > 0.05) were found between crowns made of Cerasmart 270 and LiSi Blocks. Regarding the failure types, most of the restorations showed dominantly irreparable fracture types (Figure 4A,B), whereas crown specimens (Groups 4 and 6) that have reinforced core materials of SFRC with or without fiber posts presented more repairable types (*p* < 0.05) (Figure 4C).

**Figure 5:**
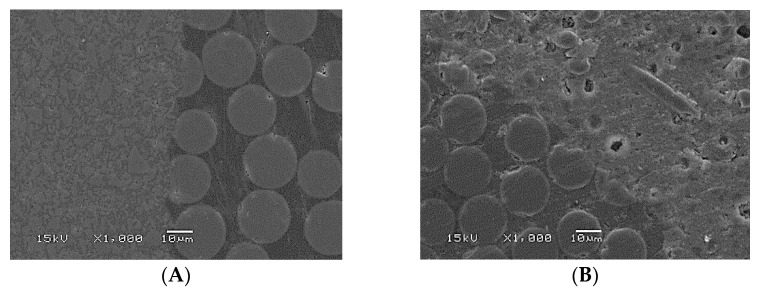

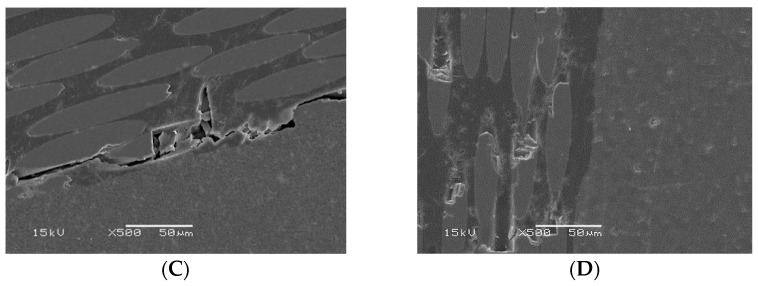
SEM images (×500 and 1000 magnification) of the adhesive interface (arrows) between fiber post and luting materials. (**A**) fiber post with Gradia Core before loading test, (**B**) fiber post with SFRC before loading test, (**C**) fiber post with Gradia Core after loading test (cracked), (**D**) fiber post with SFRC after loading test.

Representative SEM pictures of the bonding interface between fiber posts and used luting materials, before and after loading tests, are shown in Figure 5. After loading, the crack path propagated along the interface between the fiber post and the Gradia Core.

## 4. Discussion

The restoration of RCT incisors with significant tooth structure loss poses a significant clinical challenge, and choosing the right post-core system and final crown restoration may be crucial to the treatment’s success [25]. In this study, various fiber-reinforced post-core and crown materials were utilized to reinforce damaged RCT anterior teeth. Our hypotheses were rejected because fracture behavior differed significantly between the restorative techniques used (Figure 3 and Figure 4).

In this series, an attempt was made to mimic a more natural fracture behavior of the restorations by using flowable SFRC as the core material, with or without traditional (prefabricated unidirectional) glass fiber posts under the surface layer of direct and indirect crown restorations, i.e., biomimetic restorations. Biomimetics relates to the repair of affected dentition, mimicking the characteristics of a natural tooth in terms of biomechanical, functional and appearance competences [26]. The flowable SFRC (everX Flow) utilized in this research has been identified as having high fracture toughness and flexural strength [27,28]. To our knowledge, there are no other direct dental composites with fracture toughness values greater than 2.6 MPam^1/2^. As a result, we assume that SFRC-reinforced post-core systems would be able to withstand the loads needed for complete anterior crown restorations. Thus, we expected that post-core systems reinforced by SFRC could withstand the loads required for anterior teeth restorations. 

Teeth restored with traditional fiber posts (Approaches B and C) had significantly higher load-bearing capabilities than those restored without fiber posts (Approaches A and D). This finding conflicts with Garoushi et al. and Bijelic et al.’s results, which found no difference in loading resistance when anterior decoronated teeth were restored with traditional fiber posts or only SFRC posts and cores [19,29]. However, they were using experimental packable SFRC with 3 mm-long fibers, which is different from the flowable SFRC used in this research. On the other hand, our results are in accordance with earlier research in view of reinforcing the impact of fiber posts on anterior restorations [17,24,30], while others have shown that fiber posts do not reinforce teeth and actually raise the risk of catastrophic fractures [31,32].

The distribution of biting forces along the root is improved by glass fiber posts that have a close elastic modulus to dentin. In fact, the resistance of fiber posts to dislodgement is determined by their adhesion to the root dentin [15]. Therefore, the most important requirement is to achieve a durable adhesion between the composite matrix of fiber posts, luting materials, and root canal dentin.

Interestingly, the fracture load of Group 9, where SFRC was used as post-luting and core build-up resin, was the highest among all experimental groups (Figure 3). SFRC was tightly connected to the fiber post and root dentin, minimizing the drawbacks of using a weak link between them (Figure 5). Furthermore, the short fibers inside the canal were positioned correctly from a biomechanical perspective, reducing all potentially harmful tensile stresses when the restorations were loaded (Figure 5). Numerous studies have documented that high stresses can be imposed upon luting cements, particularly in the cervical area [15,16,17,18], and in vitro fatigue investigations have shown that post-luting cement microfractures or cracks are the initial failure mode that assists the development of catastrophic failure [32,33]. In the literature, the available data about fracture toughness values of various light or dual-cured luting composites ranged from 0.5 to 1.3 MPa m^1/2^, which are lower values than flowable SFRC [34,35,36,37,38].

However, the question arises as to whether the light-cured SFRC can achieve sufficient polymerization within the root canal. Previous studies by Lassila et al. and Frater et al. showed that flowable SFRC material could be polymerized properly inside the root canal, barely achieving the microhardness levels of dual-cure material [1,23]. This is due to the material’s translucency, as well as the fact that its randomly oriented fibers will conduct and scatter light over longer distances [38,39,40].

Under static loading conditions, tested CAD/CAM crown restorations showed improved load capacities compared to direct PFC composite restorations (Figure 3). This is in line with the latest results from a retrospective clinical study in Norway [8], where indirect restorations were more likely to survive the observation period than direct PFC restorations. Furthermore, according to the review study by Stavropoulou and Koidis, 63% of direct restorations and 82% of indirect restorations survived a 10-year period of observation [7]. On the other hand, a Cochrane review by Sequeira-Byron et al. [41] found no substantial difference between indirect and direct restorations. More importantly, studies have shown that the chance of RCT tooth fracture increases with a decreasing amount of remaining tooth substance [42,43].

Regarding the fracture patterns, restorations that have reinforced core composites of flowable SFRC with or without fiber posts presented more repairable types of fractures than restorations restored with Gradia as the core material. This result is in line with previous studies that showed that the SFRC core enhances the restored damaged incisors’ failure mode to be more repairable [1,19,29]. All of the repairable fractures had the core build-up and the crown separated from the tooth surface, indicating adhesive failure. When conventional fiber posts were utilized, nearly half of the failures presented as a marginal gap from the lingual surface as described by other studies [32,44]. From a clinical standpoint, this type of failure is extremely critical because it is difficult to detect at an early stage, can result in bacterial infection of the root canal system, and can lead to endodontic failure [45]. This may be due to the fiber post’s flexibility, as the post allows for considerable core movement, resulting in increased microleakage underneath the crown. Despite the fact that many specimens with indirect crowns had irreparable failure, none of the CAD/CAM-fabricated crowns showed adhesive failure, which may explain the high value of bonding achieved.

In this study, similar to other laboratory loading investigations, the fracture forces (45° loading angle) were applied evenly on the incisor margins. From a mechanical point of view, this is the most critical area of the maxillary central incisor [24].

The maximum biting forces of anterior teeth vary in the literature, but the most common value is about 200 N [46]; this is in the same range as the failure loads of specimens restored with conventional fiber posts (Approaches B and C). As a result, it is possible that incisor teeth with a 2 mm ferrule and a fiber post can withstand normal biting forces. However, the effect of fatigue loading and oral parafunctional activity such as bruxism was not considered in the current investigation.

The fracture resistance values of restored teeth measured by different investigators were reported using various measurement parameters. Initial cracking, defined as the initiation of cracks, or a load reduction by an absolute or relative amount were the parameters [1,17,29,30].

In this investigation, the ultimate quasi-static load on the final fracture was identified. Instead of being impactive and isolated in nature, stress employed to the dental restorations and teeth is generally low and cyclic. However, due to a linear relationship between fatigue and static loading, the quasi-static load test also provides valuable information about fracture behavior and load-bearing strength [47,48]. Deviations in tooth morphology are also one of the limitations of this study. Thus, further fatigue and adhesion testing studies are required to validate the findings of this laboratory research.

## 5. Conclusions

Within the limitations of this study, the following conclusions could be drawn:

1. For restoring extensively damaged anterior teeth, unidirectional fiber posts are recommended.
2. The use of flowable SFRC as post-luting and core material, with regular fiber posts, revealed promising outcomes regarding load-bearing capacity and failure modes.
3. Indirect CAD/CAM crown restorations showed improved load capacities compared to direct conventional composite restorations.

## Figures and Tables

**Table 1:** The used materials.

Brand (Code)	Manufacturer	Type	Composition
G-aenial Anterior (PFC)	GC Corp, Tokyo, Japan	Hybrid microfilled composite	UDMA, dimethacrylate co-monomers, pre-polymerized silica and strontium fluoride containing fillers 76 wt%
everX Flow (SFRC)	GC Corp, Tokyo, Japan	Flowable fiber reinforced composite (bulk shade)	Bis-EMA, TEGDMA, UDMA, micrometer scale glass fiber filler (100–300 µm and Ø7 μm), Barium glass 70 wt%, 46 vol%
Gradia Core	GC Corp, Tokyo, Japan	Dual-cured core build-up composite	Methacrylic acid ester 20–30 wt%, fluoro-alumino-silicate glass 70–75 wt%, silicon dioxide 1–5 wt%.
Cerasmart 270	GC Corp, Tokyo, Japan	CAD/CAM block	Bis-MEPP, UDMA, dimethacrylate co-monomers, silica and barium nano glass 71 wt%
Initial LiSi Block	GC Corp, Tokyo, Japan	CAD/CAM block	Not available
G-CEM LinkForce	GC Corp, Tokyo, Japan	Dual-cured, self-adhesive cement	Paste A: fluoroalumino silicate glass, initiator, UDMA, dimethacrylate, silicon dioxide. Paste B: silicon dioxide, UDMA, dimethacrylate, initiator, inhibitor
MI Core Fiber Post	GC Corp, Tokyo, Japan	Regular fiber post	UDMA, PMMA, glass fibers				

UDMA, urethane dimethacrylate; TEGDMA, triethylene glycol dimethacrylate; Bis-EMA, ethoxylated bisphenol-A-dimethacrylate; Bis-MEPP, bis (p-methacryloxy (ethoxy)1-2 phenyl)-propane; PMMA, polymethylmethacrylate; wt%, weight percentage.

**Table 2:** Different post-core and crown restorations (*n* = 10/group).

Group	Post-Core Restoration	Final Crown Restoration
1	Gradia Core as post-core	Conventional direct PFC
2	Fiber post and Gradia Core	Conventional direct PFC
3	Fiber post and SFRC core	Conventional direct PFC
4	SFRC as post-core	Conventional direct PFC
5	Fiber post and Gradia Core	Cerasmart 270 CAD/CAM
6	Fiber post and SFRC core	Cerasmart 270 CAD/CAM
7	SFRC as post-core	Cerasmart 270 CAD/CAM
8	Fiber post and Gradia Core	LiSi Block CAD/CAM
9	Fiber post and SFRC core	LiSi Block CAD/CAM
10	SFRC as post-core	LiSi Block CAD/CAM
11	Sound teeth as control	
